# Summertime dosage-dependent hypersensitivity to an angiotensin II receptor blocker

**DOI:** 10.1186/s13104-015-1215-8

**Published:** 2015-06-09

**Authors:** Donald R Forsdyke

**Affiliations:** Department of Biomedical and Molecular Sciences, Queen’s University, Kingston, ON K7L3N6 Canada

**Keywords:** Environmental temperature, Angiotensin II receptor, Losartan, Hypotension, Acute kidney injury, J-curve

## Abstract

**Background:**

Summertime dips in blood pressure (BP), both in normotensive and hypertensive subjects, are well known. However, the dips are small and are not related to particular forms or doses of antihypertensive medication. Nevertheless it is the practice in some quarters to decrease antihypertensive medication in summer, and/or to increase in winter. Large scale studies being inconclusive, there are calls for long-term examination of the relationship between environmental temperature and blood pressure in single individuals under medication.

**Case presentation:**

While analyzing data from a subject whose BP had been controlled for a decade with the angiotensin-II receptor blocker losartan, an extreme, dosage-dependent, summertime dip came to light. Downward dosage adjustment appeared essential and may have prevented hypotension-related pathology.

**Conclusion:**

The benefits of aggressive medication (the “J curve” phenomenon) being debated, the possibility of seasonal hypersensitivity, perhaps explicable in terms of differential signaling by countervailing receptors, should be taken into account when considering dosage adjustments in hypertensive subjects.

## Background

### Conflicting opinions on seasonal dosage adjustment

Small summertime declines in blood pressure (e.g. 5–10 mm Hg) have long been known, both in normotensive and hypertensive subjects. Indeed, adverse cardiovascular events being more frequent in winter, it is the practice in some quarters to decrease antihypertensive medication in summer, and/or increase in winter [1]. But this is controversial. Ambulatory blood pressure (ABP) recordings often reveal a small dip in BP values when subjects are resting at night [2]. In Italy, Modesti et al. reported in 2006 [3] that in summer this night-time dipping was less evident, and systolic values (SBPs) were slightly *increased*; it was only with day-time BP measurements that a summer decrease was evident. They cautioned against reducing dosage of antihypertensive medication in hot weather. On the other hand, based on clinic BP measurements of 500,000 subjects drawn from ten climatically diverse regions of China, in 2012 Lewington et al. [4] affirmed that “higher doses or additional drug(s) may be required in winter to achieve the same blood pressure control as at other times of the year”. In other words, they advised lowering dosage in summertime. Indeed, in 2013 Modesti came to agree that “it is possible that heat-exposed subjects need lower dosages… because of lower BP in warm conditions” [5].

However, seasonal influences on responses to specific antihypertensive medications are not well documented. Apart from logistic considerations (e.g. patient confidentiality), this may be due the relative newness of some medications, so that long-term studies are not yet available. In Japan, Hozawa et al. [6] relied on the home BP measurements of volunteers, but had no information on medications. In Scotland, Aubinière-Robb et al. [7] relied on clinic measurements of treated hypertensive subjects, but had “incomplete prescribing data”. Furthermore, their location implied more concern for potential adverse effects of increases in BP in cold weather, than of decreases in BP in hot weather. The subtropical island of Taiwan has temperatures closer to those in summer-time Ontario, but the winter-summer variation is much less. For Taiwan, Tu et al. [8] reported no influence of season on the response to antihypertensive medication, but type and dosage were unspecified.

The most definitive study to date is the above noted work of Lewington et al. [4]. The *percentage* differences between summer and winter did not differ between those on antihypertensive medication (type unspecified), and those who were not. However, *absolute* differences were greater in hypertensive subjects (differences averaging 11.0 versus 9.6 mm Hg). Floras has recently cautioned that when marginal hypertension is diagnosed in summer-time, initiating therapeutic dosages may be suboptimum, but the possibility of extreme seasonal variation in sensitivity to medication was not entertained [9].

### Calls for long-term single patient studies

Despite many studies, seasonal variations in BP are not clearly related to particular forms or dosages of medication in individual subjects. It is recognized that “patients are exposed to antihypertensive treatment for decades; yet, long-term safety of these drugs is not well-reported. Most prospective randomised trials end after a few years without long-term follow up” [10]. Indeed, in 2013 Modesti et al. [11] declared that some of the limitations of their approach “would be addressed in future studies based on repeated measurements according to a longitudinal design and focusing on the assessment of temperature and BP changes within a single individual”. This need for long-term single-individual studies was echoed in 2012 by Cuspidi et al. [12], and in 2013 by Tomlinson et al. [13], who called for “carefully designed studies using individual level patient data to examine this issue in more depth”. To some extent, the present study meets this requirement, but regrettably with the absence of night readings. In 2011 Handler [14] reported a case where the subject, based on home BP readings and postural hypotension, had opted to stop medication in summer, but there were few details. In 2013 Chen et al. [15] reported a 3 year follow-up of hypertensives treated with the angiotensin converting enzyme (ACE) inhibitor (benazepril); average seasonal fluctuations were of the same order as reported by Lewington et al. [4]. It was concluded that “patients should monitor and treat blood pressure more carefully in cold days”.

### J-curve phenomenon

Seasonal BP variations are not seen as related to the so-called “J curve” phenomenon [16]. While the benefits of decreasing blood pressure are clear, there comes a point below which there are negative consequences, marked by a J-like inflection on plots of adverse cardiovascular events against BP. Such consequences include acute kidney injury, now becoming more evident among those on medication [13]. Indeed, it is held that its “important implications for clinical practice should make investigation on the J-curve phenomenon a priority for cardiovascular medicine” [16].

While analyzing data from a subject whose BP had been controlled for a decade with the angiotensin-II receptor blocker (ARB) losartan, an extreme, potentially dangerous, summer-time influence came to light [17]. ARBs being treatment of choice for millions of subjects, it is unlikely this is an isolated case.

## Case presentation

### Materials and methods

In August 1999, mild hypertension (circa 150/90 mm Hg) was found during routine examination of a 60 year old biomedical researcher. In the 1960s he had been briefly involved in hypertension research^a^. When studying the activation of cultured human lymphocytes in the 1980s he discovered a gene (*RGS*-*2*) [18] that was later found to regulate BP [19, 20]. With appreciation of possible immunological aspects of hypertension [21], he followed the course of his new condition with deep professional interest. This led to his carrying out all readings for, and authoring, the present report. Beginning in January 2000, resting BP readings were taken at least once daily (usually both in early morning and late evening) by the subject at his home. The continuing accuracy of his Omron digital BP monitor (model HEM-712C) was ascertained by comparing with readings from his mercury sphygmomanometer, with those obtained in his physician’s office^b^ and, in 2015, by comparing with a new Omron monitor (HEM-7121C).

Since Ontario Climate Centre records of daily temperatures for the subject’s lakeside city (Kingston, Ontario) did not become available until 2008, values for a location 24 km north (Hartington) were employed. The latter tends to be 2–3° cooler/hotter in winter/summer than Kingston. In the period of this study, indoor temperatures were regulated at around 22°C during cold weather. In summer months fans were employed and only short periods were spent in air-conditioned environments.

Throughout the study period standard blood and urine tests remained within normal ranges, except that on occasions creatinine levels approached high normal. The subject’s resting pulse had registered around 50/min for many years. His lifestyle was that of an academic workaholic—several hours a day at a computer interrupted by frequent brisk walks, and twice weekly runs (2 km). Height and weight had remained relatively constant throughout adult life (currently 1.76 m and 72 kg; BMI = 23.2). The hypertension was assumed to be primary (‘essential’), and was not further investigated. However, at an early stage antihypertensive medication was associated with postural hypotension and an instance of acute renal colic. These encouraged close home BP monitoring with dosage adjustment by the subject targeting 130/80 mm Hg. While the present report is primarily concerned with a 12 year period when losartan was the sole medication (2003–2014), the stage will be set with a brief account of an initial 3 year exploratory period with various other medications.

### The period 2000–2002

**Figure 1:**
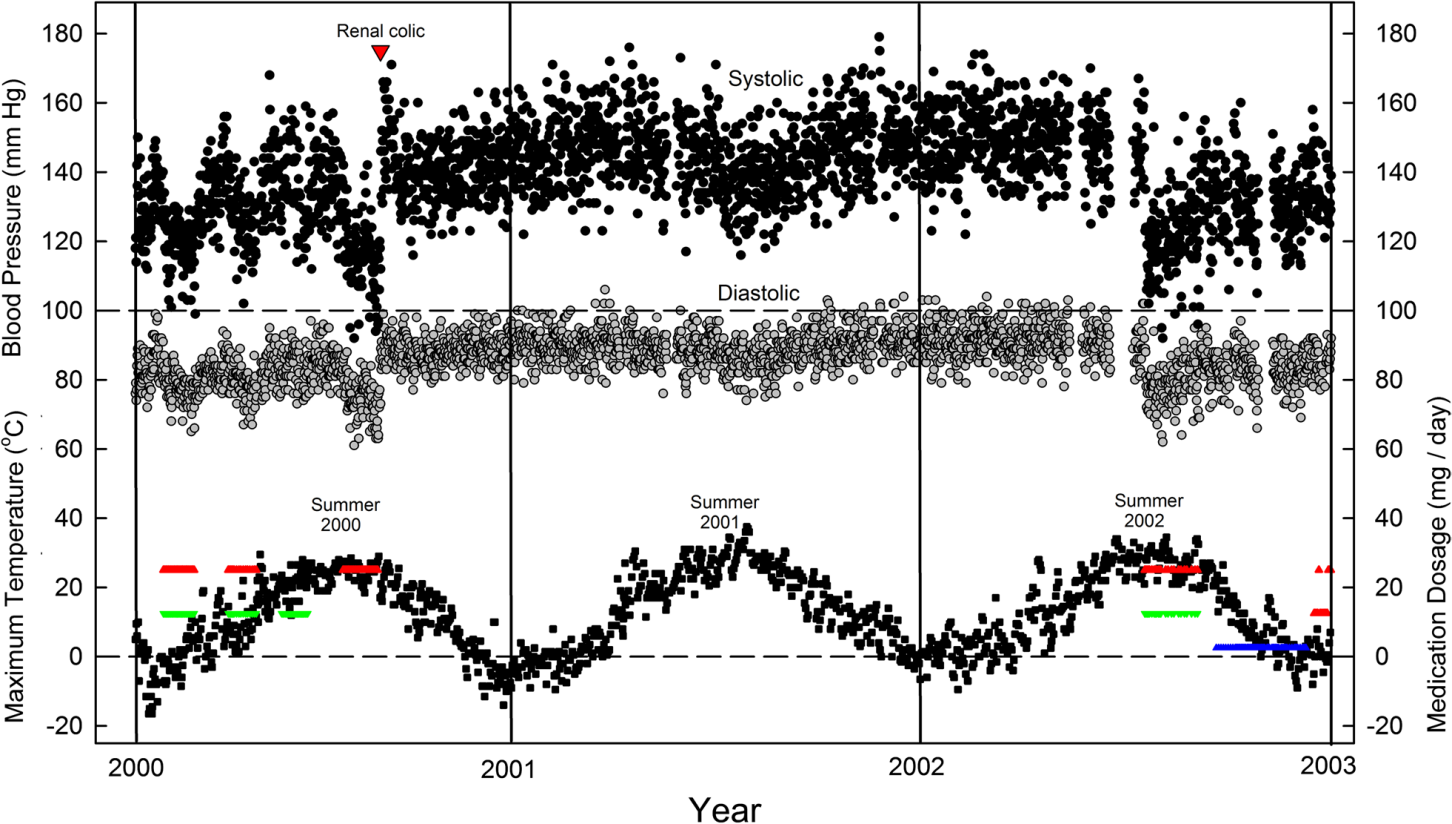
Daily variation in SBP (*black circles*) and DBP (*grey circles*) over a 3 year period, as related to (1) maximum daily environmental temperatures (*small black squares*) and (2) periodic treatments at constant dosage with losartan (*red triangles*), chlorothiazide (*green triangles*) or ramipril (*blue triangles*). At *bottom right*, the two rows of *red triangles* mark the initiation of a 12 year period (2003–2014) where the day-to-day dosage of losartan was varied. Renal colic in the year 2000 is marked by a large *red triangle*. At that time home BP measurements were usually taken 2–3 times a day—in the early morning, in the early afternoon, and in the evening. All measurements are directly plotted. *Gaps* indicate periods of travel when readings were discontinued. Since records of temperature values for the subject’s lakeside city (Kingston, Ontario) did not become available until 2008, values for a location 24 km north (Hartington) were employed.

In the year 2000, two one-month trials (Feb., Apr.) of daily losartan (25 mg) with chlorothiazide (12.5 mg) resulted in progressive falls in day-time BP, with some systolic values (SBP) around 100 mm Hg (Figure 1). Consistent with this, the subject experienced some dizziness on standing up abruptly. On cessation of these medications, BP values progressively returned to previous levels.

In June, chlorothiazide alone (12.5 mg) had little effect. However, losartan alone (25 mg), taken at the height of summer (August, with environmental temperatures approaching 30°C), produced a progressive and more profound fall in pressure, with SBP values again below 100 mm Hg, and diastolic (DPB) values approaching 60 mm Hg. Shortly after cessation of therapy there was acute renal colic and blood pressure rose abruptly (Figure 1). A ureteral stone observed on X-rays was presumed to have passed in the urine.

**Figure 2:**
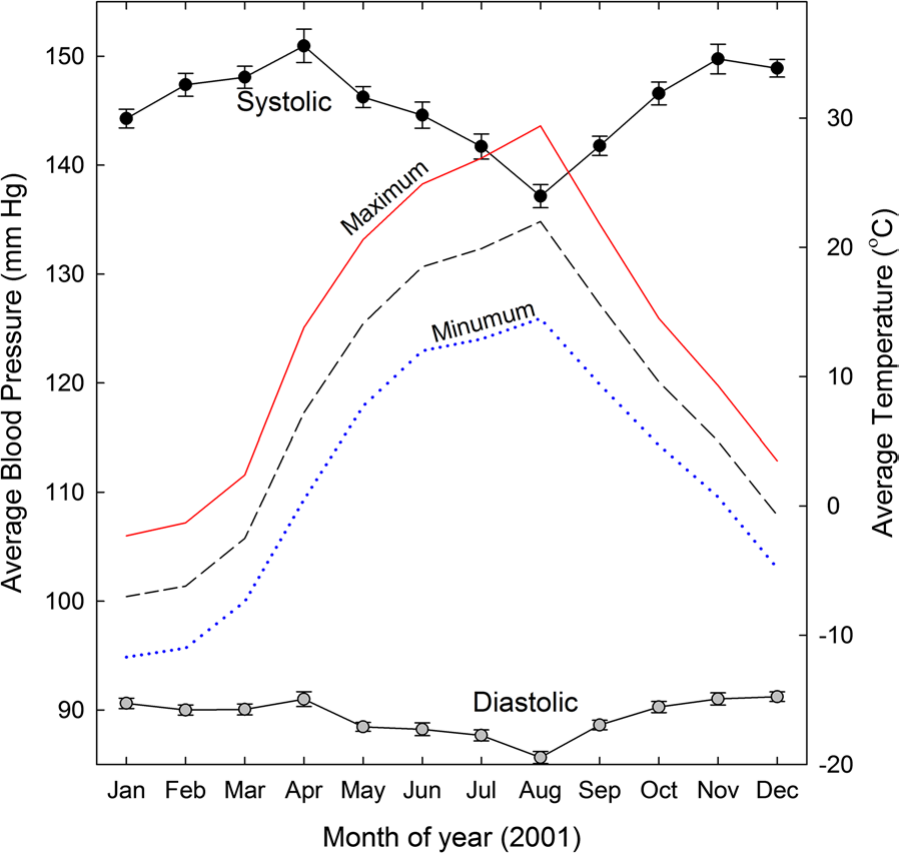
Seasonal variation in SBP and DBP in absence of hypertensive medication. Daily values for each month in the year 2001 are averaged and plotted with standard errors. Corresponding monthly average temperature values are shown without symbols (maximum, *continuous red line*; average, *dashed black line*; minimum, *dotted blue line*).

In view of the timing, and the subject not having previously experienced renal colic, it was considered likely that stone formation had been facilitated by hypotension. Indeed, there is now increasing awareness that acute kidney injury (AKI) can follow ARB medication in a range of settings, particularly during acute hypovolemic illness [13]. Medications were avoided for the next 2 years and pressure values remained relatively constant in the 150/90 range. In the summer of 2001 there was the expected small BP dip, which correlated inversely with environmental temperature (Figure 2).

In the summer of 2002 mild hypertension was confirmed by one session of 24 h ambulatory BP (ABP) monitoring and, for a 6 week period (beginning in the last week of July), daily combination therapy with losartan (25 mg) and chlorothiazide (12.5 mg) was resumed (Figure 1). Again, there were extreme declines in pressure values and some minor dizzy episodes. BP was controlled more satisfactorily with the ACE inhibitor, ramipril (2.5 mg/day; late September to early December). However the subject experienced a persistent dry cough, so ramipril was discontinued. Although there had been some dry coughing with losartan, therapy with losartan alone was resumed at the end of December 2002 (rows of red triangles at extreme right in Figure 1). Dosage was adjusted daily by the subject according to his BP readings. This proved satisfactory for the next 12 years, despite some dry coughing.

### The period 2003–2014

With various combinations of half (12.5 mg) and whole (25 mg) tablets, daily losartan dosage was varied over the range, 0, 12.5, 25, 37.7 and 50 mg, taken either in the early morning or, from December 2010 onwards, split between mornings and evenings (under guidance of BP readings taken at the same times). Further fine adjustment was attempted by trying to maintain regular dosage patterns—e.g. 12.5, 25, 12.5, 25 mg, etc. Apart from weekly sildenafil citrate (50 mg), which tended to lower BP, losartan was the sole medication.

**Figure 3:**
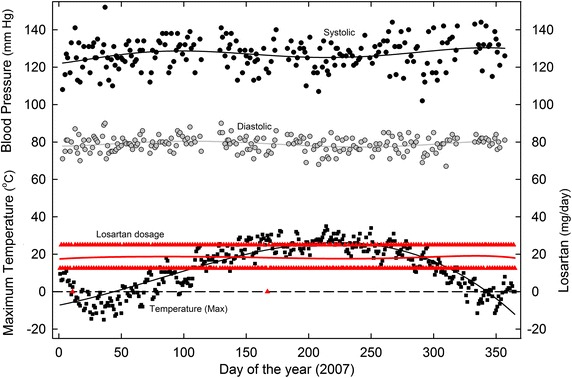
Daily variation in SBP and DBP in 2007, as related to (1) maximum daily environmental temperatures and (2) varying losartan dosage. Home BP measurements were usually twice daily—in the early morning and late evening—and these values were averaged for plotting. Least-squares regression (sixth order) polynomial fits to the points are shown as *continuous lines* (the fit is third order for the *black temperature line*). For other details see Figure 1.

For the first 4 years (2003–2006) the required average losartan dose was 16 mg/day, rising to 18 mg/day for the next 3 years (2007–2009). Thereafter, the average requirement rose from 19 mg/day (2010) to 44 mg/day (2013) and 33 mg/day (2014). An example of the ability to fine-tune day-time BP readings over the 2003-2009 period is shown for the year 2007 (Figure 3). With relatively constant losartan dosages (average 18 mg/day) blood pressure readings were maintained at acceptable values (130/80 mm Hg). There was generally no need for special dosage adjustments in the hot summer season.

**Figure 4:**
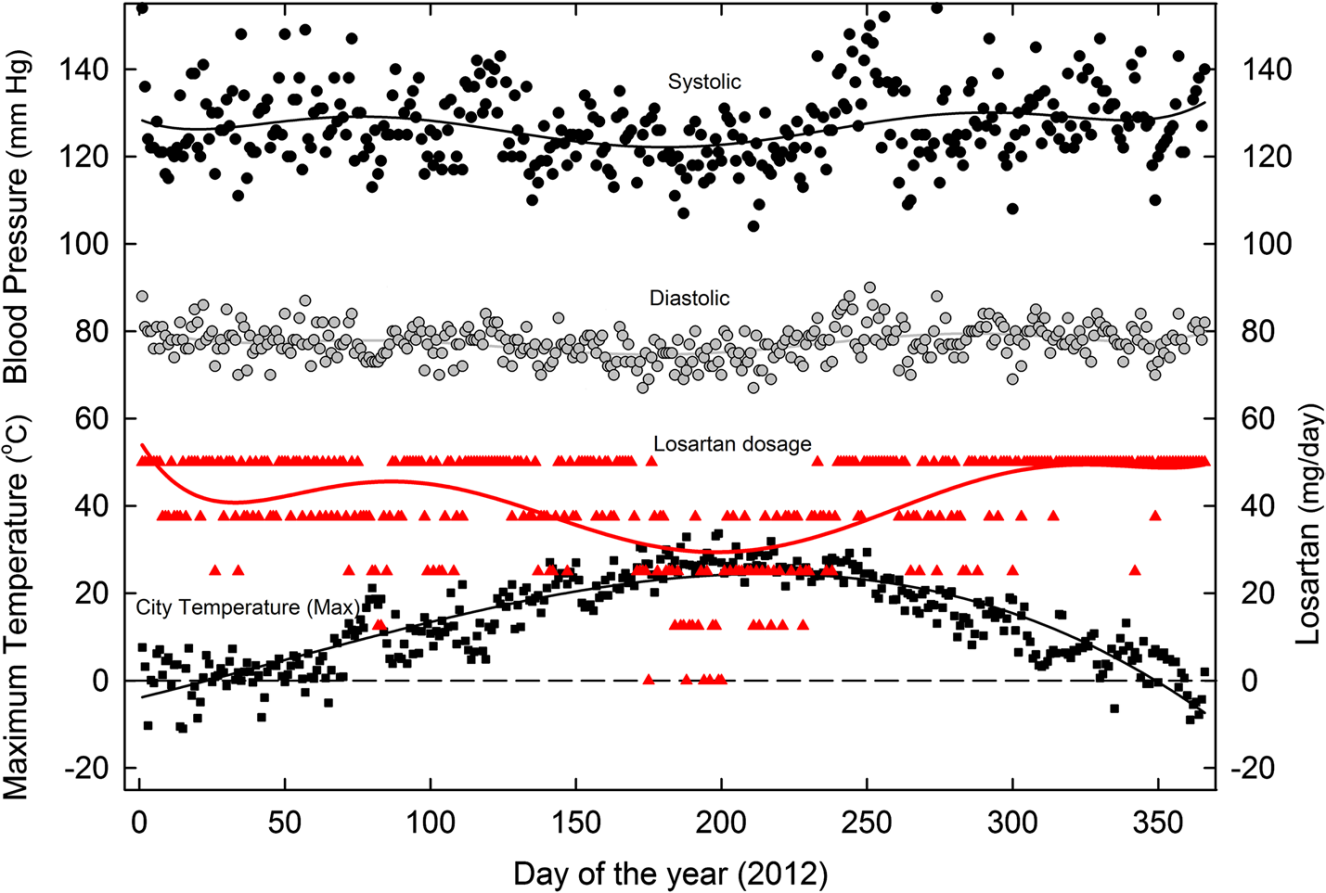
Daily variation in SBP and DBP in 2012, as related to (1) maximum daily environmental temperatures and (2) varying losartan dosage. For details see Figures 1, 3.

These same BP values were sustained in the 2010–2014 period. However, when, for some unknown reason, the total losartan requirement *increased*, an extreme *downward* dosage adjustment became necessary in the summer season. This is shown for the year 2012 in Figure 4.

**Figure 5:**
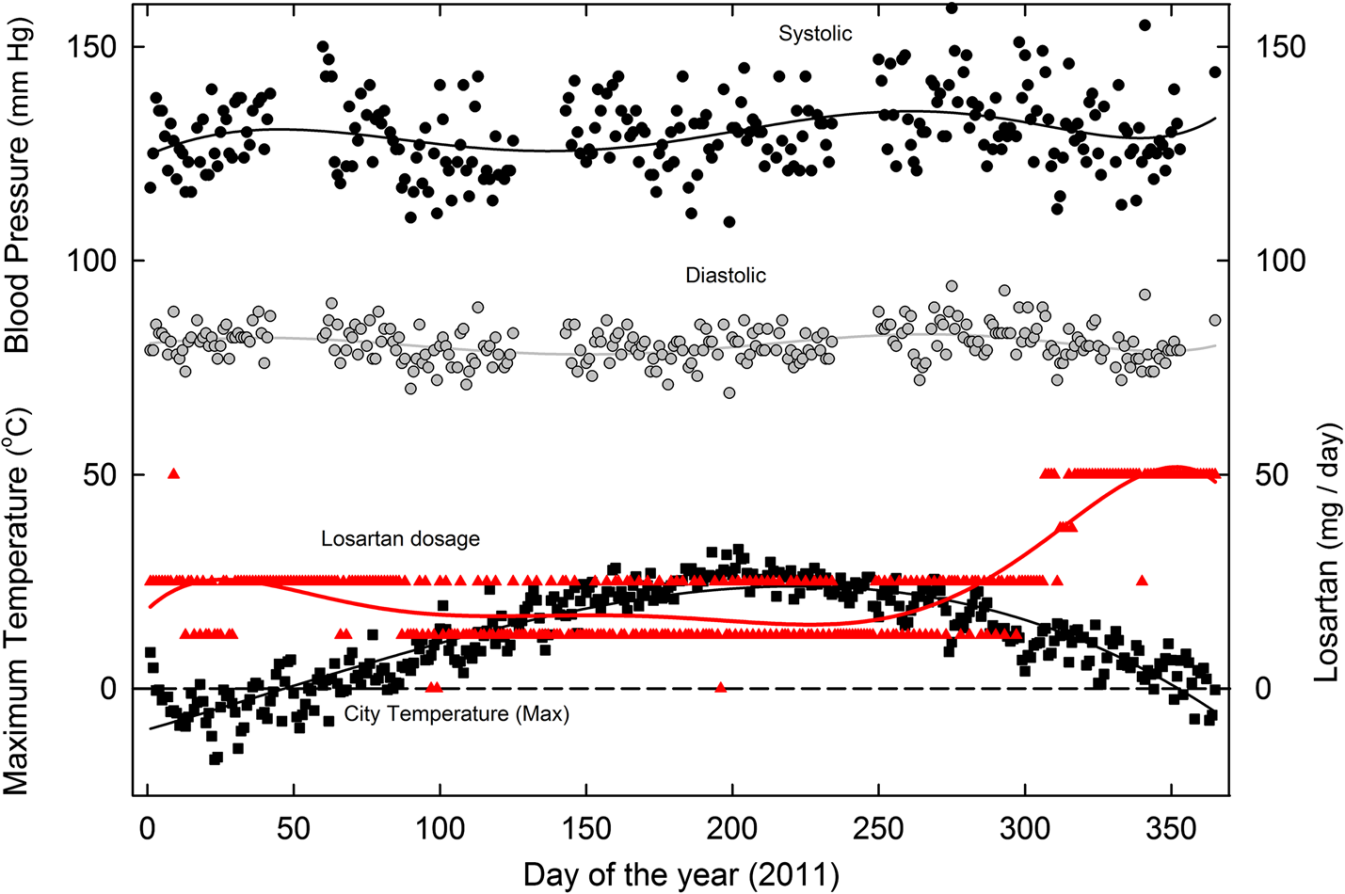
Daily variation in SBP and DBP in 2011, as related to (1) maximum daily environmental temperatures and (2) varying losartan dosage. For details see Figures 1, 3.

The detailed BP plot for 2011, when increased losartan dosage first became necessary, is of special interest (Figure 5). BP levels were maintained relatively constant by *decreasing* losartan dosage in the summer months and *increasing* dosage in the following winter. The sub-zero maximum daily temperatures early in the year were associated with 25 mg/day dosages. The increase in losartan requirement to 50 mg/day began in the late fall when maximum temperatures were still above zero, so seeming to reflect an influence internal to the subject, as well as from the environment.

**Figure 6:**
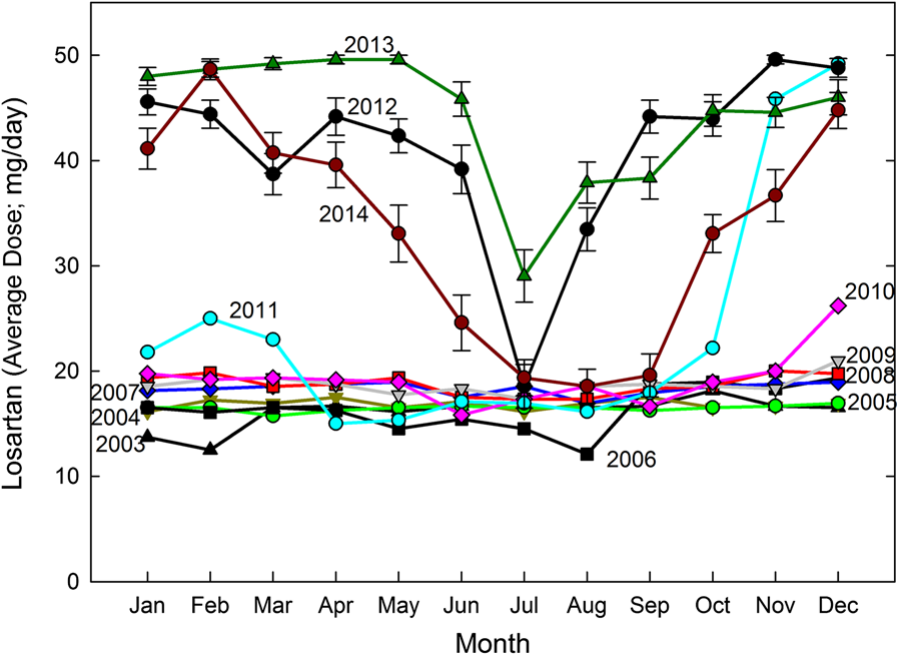
Monthly losartan requirements for a 12 year period (2003–2014). 2003, *black triangles*; 2004, *dark yellow triangles*; 2005, *green circles*; 2006, *black squares*; 2007, *blue diamonds*; 2008, *orange squares*; 2009, *grey triangles*; 2010, *red diamonds*; 2011, *cyan circles*; 2012, *black circles*; 2013, *green diamonds*; 2014, *dark red circles*. Data for 2012–2014 include standard errors.

Monthly losartan requirements for the entire 12 year period are shown in Figure 6. At the doses employed between 2003 and 2009 (average 16–18 mg/day), usually no seasonal adjustment was needed. In 2006 (a particularly hot year), only minor adjustment was needed. The plot for 2011 was distinctive. At first the requirement was high, but decreased to previous values during spring and summer. However, later in the year as environmental temperature declined, there was a sharp increase in losartan requirement. Subsequently (2012–2014), a summer requirement for extreme downward dosage adjustment emerged.

### Relationship between temperature and BP

**Figure 7:**
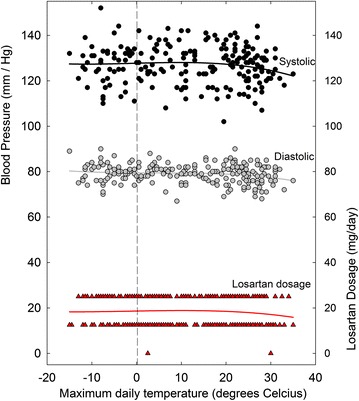
Daily values for SBP, DBP, and losartan dosages for the year 2007, as a function of the corresponding maximum environmental temperatures. Least-squares regression fits (third order polynomial) to the points are shown as *continuous lines* (*red* for losartan dosage). This is a re-plot of the data of Figure 3.

**Figure 8:**
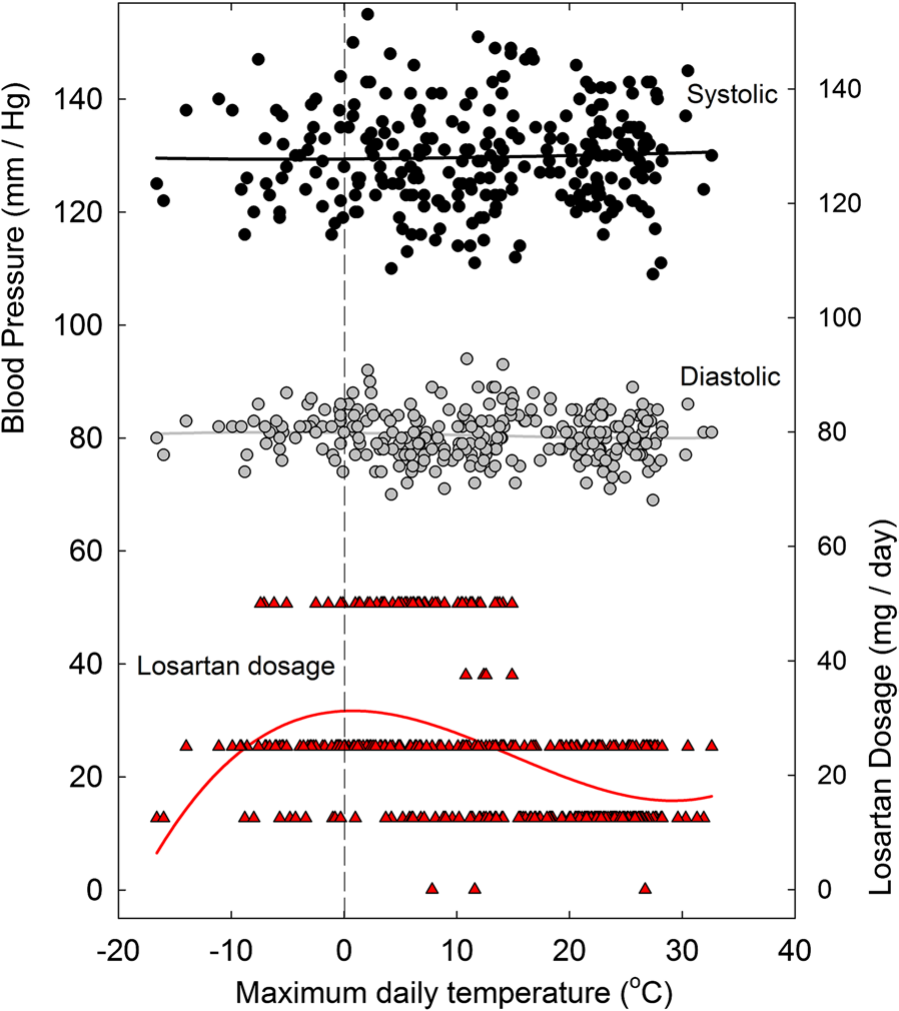
Daily values for SBP, DBP, and losartan dosages for the year 2011, as a function of the corresponding maximum environmental temperatures. Least-squares regression fits (third order polynomial) to the points are shown as *continuous lines*. This is a re-plot of the data of Figure 5.

Plots of BP and losartan dosage against temperature showed minimal influence of temperature during the 2003–2009 period. Figure 7 shows data for 2007 (from Figure 3). The regression line for losartan dosage (red) was essentially horizontal. However, plots for the year 2011 (Figure 8) showed a biphasic linear regression fit to losartan dosage. The ascending limb of the regression reflects the dosage increase from 25 mg/day in the cold early part of the year, to 50 mg/day in the less cold late part of the year. The descending limb of the regression reflects the decreasing requirement during the summer months.

**Figure 9:**
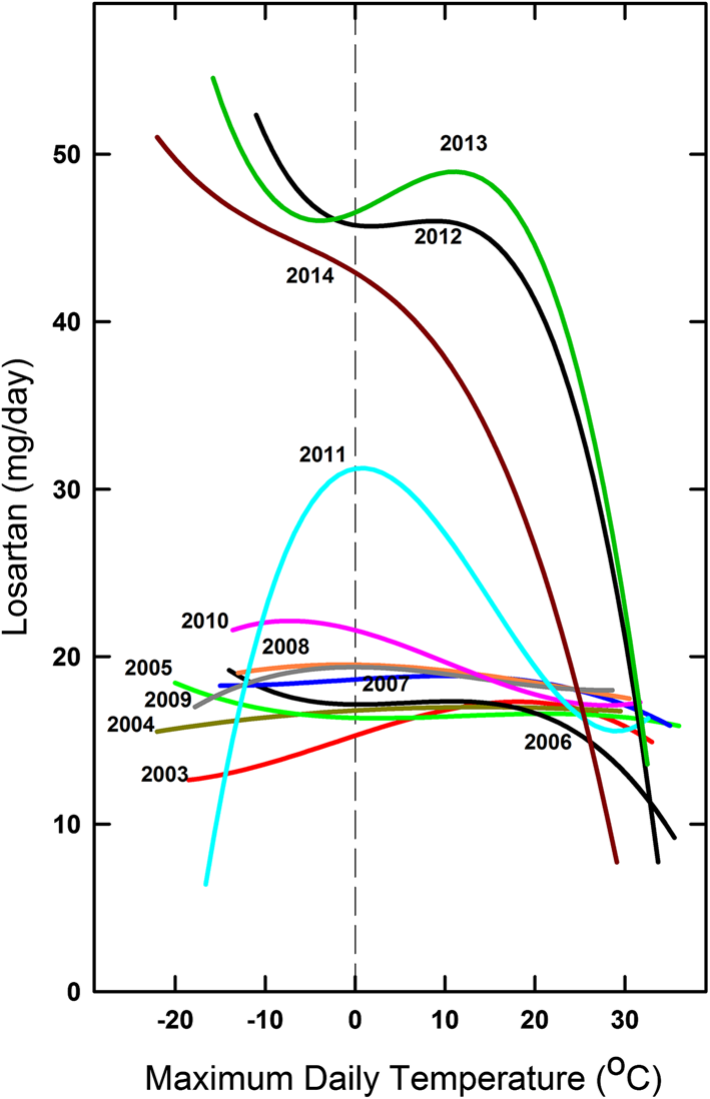
Relationship between daily losartan requirements for the 2003-2014 period and corresponding maximum environmental temperatures. The third order regressions through data points (e.g. Figures 7, 8) are shown for each year. *Line colouring* for different years follows that of Figure 6. Consecutive r^2^ values for 2011–2014 were 0.22, 0.38, 0.34, and 0.45.

Figure 9 shows regression plots for the entire 2003–2014 period. The curves were essentially horizontal for 2003–2009. Thus, dosage was independent of temperature. In 2010 came the first indication of the extreme seasonal influence—explicit from 2012 onwards. Indeed, by extrapolation, under these conditions losartan could have been abandoned at around 34°C.

## Discussion

The present study arose from the belief that an interference with physiological homeostatic controls, which was deemed necessary for management of primary hypertension, would require close BP assessment—an assessment which would be facilitated by the digital devices that had become available for home-monitoring. Whether it is the actual BP level, or variation in that level, that is most responsible for adverse clinical consequences, is much debated [9, 22]. Here, the day-to-day adjustment of losartan dosage to observed *daily* BP levels would seem to address both factors.

### Angiotensin II receptors

Antihypertensive, dosage-dependent, effects of losartan were evident in early short-term studies with both normal volunteers and patients [23]. Thus, Gottlieb et al. [24] noted in 1993 that vascular dilation and BP-lowering effects were maximal with 25 mg/day and *declined* at higher doses, whereas effects deemed ‘neurohormonal,’ such as increased levels of renin and of the circulating angiotensin II octapeptide (Ang II), continued to increase at higher concentrations. With the present subject, summer losartan hypersensitivity became most evident when dosage increased from around 25 to 50 mg/day, consistent with a neurohormonal influence.

Cell surface Ang II receptors (subtypes AT_1_R and AT_2_R) are present in various mammalian species. It is the reaction of Ang II with AT_1_R, the dominant high-affinity receptor, that is blocked with high specificity by losartan [25]. Independently of losartan, the reaction normally triggers G_q_-protein signalling that mobilises intracellular Ca^++^, resulting in increased vascular tone. Such signalling is itself susceptible to modulation by regulatory factors—such as Regulator of G-Protein Signaling-2 (RGS-2) [18–20]—which are themselves subject to regulatory inputs. So determining how seasonal factors feed into this system, and whether the key seasonal factor is, indeed, temperature [11, 26], are unlikely to be easy.

Although bound to plasma albumin, losartan itself is rapidly degraded to a longer-lived, pharmacologically more potent, carboxylic acid derivative, also bound to albumin; this sustains AT_1_R blockade non-competitively for many hours [27, 28]. Thus, provided a sufficient dose is employed, and the period between doses is not too long, successive losartan doses may act cumulatively. This is consistent with the observation that, in the *spring* of 2000, following the implementation, and then cessation, of losartan therapy, BP values fell progressively, and rose progressively, each over several days (Figure 1). However, when losartan was restarted in the *summer* of 2002, the fall was immediate. This hinted at an additional seasonal influence, *conditional* on losartan dosage that would be uncovered (“unmasked”) in later years (Figures 6, 9).

### Unmasking of angiotensin II subtype 2 receptors

Treatment with ACE inhibitors *lowers* the circulating concentration of Ang II, so decreasing its reaction with the dominant AT_1_R subtype, and thus lowering BP. However, the *increase* in the circulating concentration of Ang II, following blockage of the AT_1_R subtype with losartan, should suffice to affect the losartan-insensitive, low abundance, AT_2_R subtype. Activation of AT_2_R usually *counteracts* the effects of AT_1_R activation (e.g. vasodilation not vasoconstriction) [29, 30]. It is reported for hypertension-prone rats that Ang II will cause AT_2_R-mediated vasodilation, *provided* AT_1_R is blocked and AT_2_R expression is upregulated [31, 32]. Thus, activation of AT_2_R is *conditional*, and is described as being “unmasked” or “trumped” when AT_1_R-mediated effects are inhibited by agents such as losartan [33–35]. Indeed, Abdulla and Johns [36] recently reported for rats that losartan increased the fall in BP following the AT_2_R receptor-associated inhibition of renal sympathetic nerve activity, which was part of the homeostatic response to total body fluid volume expansion, such as normally occurs in humans in summertime [37]. They concluded that: “The basal level of central AT_2_ receptor activation is not involved in the normal renal sympatho-inhibition due to volume expansion, unless the counter-regulatory AT_1_ receptors are blocked”. Thus, there is again an “unmasking” effect of losartan. These conclusions from rodents are supported by studies of Bartter and Gitelman syndromes (BS/GS) patients, where there is endogenous antagonism of AT_1_R signalling that in many respects resemble inhibition by losartan (e.g. Ang II elevation) [38, 39].

### Hypothesis

A working hypothesis, consistent with animal experiments and BS/GS studies, is that under conditions of heat-stress (e.g. vascular dilation, salt loss), there is increased expression of a countervailing, losartan-insensitive, receptor subtype (AT_2_R). By *lowering* BP in response to antiotensin-II, AT_2_R would facilitate fine-tuning of the AT_1_R-mediated vasoconstriction that supports BP when superficial veins dilate to enhance body cooling. This AT_2_R activity might be sufficient to explain a small summertime BP dip found in normal human subjects whose Ang II levels are not increased (Figure 2). The dip would be greatly enhanced when Ang II levels are increased at higher losartan dosages. Under this condition, the excess of Ang II would be expected to react with the AT_2_R, so greatly amplifying the losartan-induced fall in BP. To this extent, the present human study is supportive of most rodent and BS/GS studies. The hypothesis predicts that summertime dips would be decreased either by ACE inhibitors, or by AT_2_R antagonists, such as EMA401 [40]. Indeed, the study of benazepril by Chen et al. [15] supports the hypothesis and is in keeping with the recommended caution regarding combination therapy with ACE inhibitors and ARBs [41]. AT_2_R agonists, including truncated Ang II fragments, are possible novel antihypertensive agents [34].

### Night-time dipping

ABP recordings often reveal a small dip in BP values when subjects are resting at night. Although carried out on biased groups (members of different summer and winter populations that had been selected to attend hypertension clinics), ABP studies in Italy [2, 3] found that, in summertime, night-time dipping was less evident and SBPs were slightly *increased*; it was only with day-time BP measurements that the summertime decrease was evident. The night-time SBP increase was particularly apparent in elderly subjects receiving antihypertensive medication (type not specified). While noting that “milder sleep problems associated with hot weather cannot be completely excluded” [3], and that there may be “different sleeping behaviors between summer and winter” [2], the authors suggested that there is often a, clinic-directed or self-directed, reduction in medication in summertime, either because of a measured daytime lowering of BP, or because it is “common knowledge” that such lowering would have occurred [2]. Thus, those who would reduce the number of medications, or reduce dosages (as in the present study), were cautioned by Modesti et al. [3] that “the results of our study clearly indicate that the practice of reducing treatment in the summer in the elderly based on low clinic BP values is not good, because it might be responsible for a potentially dangerous increase in night BP”.

Nevertheless, given that hypertension-related adverse cardiovascular events are less in summer, then correcting, at this time of the year, for the daytime *decrease* in SBP, may be more important that correcting for a night-time *increase*. Determining the swings and roundabouts of this is a matter for future study, but a prudent interim measure might be to take some or all of whatever medications are deemed necessary in hot weather, late in the evening. Such a season-tailored ‘chronotherapeutic approach’ [12, 42] touches on the issue of the period of bioavailability of a medication after ingestion (as discussed above [27, 28]).

## Conclusions

There should be greater awareness that the inflection points on J-curves [43] might vary on a seasonal basis. This awareness should encourage close self-monitoring of BP, with appropriate adjustment of medication dosage, especially in the case of losartan. Such dosage adjustment may be necessary for those living in, or travelling to, geographical regions where temperatures are seasonally or continually high. Assuming temperature to be primary, then this caveat might also apply to those engaging in hot activities (e.g. Turkish baths, hot yoga). More comprehensive softwares in BP monitoring devises might take into account both environmental temperatures and recent BP readings, and automatically recommend daily medication adjustment. Although randomized, double-blind, trials, may sometimes lead to proposals for increases in losartan dosages (e.g. Konstam et al. [44] advise elevation from 50 to 150 mg), it would seem that the climate of the country where such trials had taken place should be considered when assessing the risk-benefits of such regimens. Indeed, hypertension has been invoked as a factor explaining higher mortality in cold climates [45]. Sadly, studies of ARB-induced hypotension and AKI, both in the elderly [46], and in diabetic patients [47], give little weight to seasonal factors and dosage variation. An association of medication-induced hypotension with cognitive impairment in the elderly has led to disparagement of “one size fits all” approaches to therapy [48]. Finally, as noted by Verberk et al. [49] there may be direct economic benefits to health care systems if excessive dosages of costly medications are avoided.

## Endnotes

^a^ This paper is dedicated to W. Stanley Peart and James F. Mowbray, whose mentorship in times long past guided me to the road ‘less travelled by’.

^b^ The office of David J. Hemings.
